# Nanotechnology Advances in the Detection and Treatment of Lymphoid Malignancies

**DOI:** 10.3390/ijms25179253

**Published:** 2024-08-26

**Authors:** Francesco Maria Adamo, Filomena De Falco, Erica Dorillo, Daniele Sorcini, Arianna Stella, Angela Esposito, Roberta Arcaleni, Emanuela Rosati, Paolo Sportoletti

**Affiliations:** 1 Department of Medicine and Surgery, Institute of Hematology and Center for Hemato-Oncology Research (CREO), University of Perugia, Santa Maria della Misericordia Hospital, 06132 Perugia, Italy; 2 Department of Medicine and Surgery, Biosciences and Medical Embryology Section, University of Perugia, 06132 Perugia, Italy

**Keywords:** lymphoid malignancies, nanoparticles, precision medicine

## Abstract

Lymphoid malignancies are complex diseases with distinct biological behaviors, clinical presentations, and treatment responses. Ongoing research and advancements in biotechnology enhance the understanding and management of these malignancies, moving towards more personalized approaches for diagnosis and treatment. Nanotechnology has emerged as a promising tool to improve some limitations of conventional diagnostics as well as treatment strategies for lymphoid malignancies. Nanoparticles (NPs) offer unique advantages such as enhanced multimodal detection, drug delivery, and targeted therapy capabilities, with the potential to improve precision medicine and patient outcomes. Here, we comprehensively examine the current landscape of nanoconstructs applied in the management of lymphoid disease. Through a comprehensive analysis of preclinical studies, we highlight the translational potential of NPs in revolutionizing the field of hematological malignancies, with a specific focus on lymphoid neoplasms.

## 1. Introduction

Lymphoid malignancies refer to a group of blood tumors affecting the lymphatic system and are characterized by a complex mutational landscape and a heterogeneous clinical course [1,2]. This biological complexity is mirrored by the broad spectrum of distinct diseases arising from lymphoid cells, commonly referred to as leukemias and lymphomas.

The advent of precision medicine and targeted therapies has drastically improved outcomes for patients with lymphoid malignancies. The diagnosis of lymphoid neoplasms has reached high levels of sensitivity, specificity, and accuracy in recent years. However, one of the major challenges in the detection of such neoplasms relies on the identification of low numbers of residual cells after treatments, which can be difficult to detect with traditional diagnostic tests. The availability of assays with high sensitivity and specificity is crucial for measurable residual disease (MRD) monitoring, as this allows for the assessment of therapeutic efficacy, the prediction of relapse risks and the identification of patients who may require therapeutic intensification or switch to different drug regimens [3]. Targeted agents include monoclonal antibodies, small inhibitors targeting oncogenic signaling pathways, immune checkpoint inhibitors, and chimeric antigen receptor (CAR) T-cells. Although these therapeutics have shown great effectiveness in treating lymphoid neoplasms, challenges remain, including the development of resistance to therapy and the risk of long-term side effects [4].

Nanoparticles (NPs) represent a promising frontier in hemato-oncology research, with potential applications in the detection and treatment of lymphoid malignancies. Through their unique physicochemical properties and customizable surface functionalities, NPs could overcome some limitations in diagnostics and therapy. NPs serve as a versatile tool for accurate detection, staging, and disease monitoring, enabling timely intervention and personalized treatment strategies [5]. Furthermore, NPs can enhance the efficacy and tolerability of conventional treatments. NPs allow the selective delivery of therapeutic agents to tumor tissues to minimize off-target toxic effects. Additionally, the multifunctional and theranostic features of NPs could combine both diagnostic and therapeutic capabilities, leading to more efficient disease management [6].

In this review, we provide a comprehensive overview of the current state-of-the-art in the application of NPs to lymphoid malignancies. We describe various types of NPs employed in nanomedicine, as well as the strategies for NP-based detection, staging, and monitoring of lymphoid malignancies. Additionally, we examine the approaches for NP-based drug delivery, immunotherapy, and active targeting through ligand-receptor interactions.

## 2. Types of Nanoparticles Applied in the Biomedical Field

### 2.1. Carbon Nanoparticles

**Figure 1:**
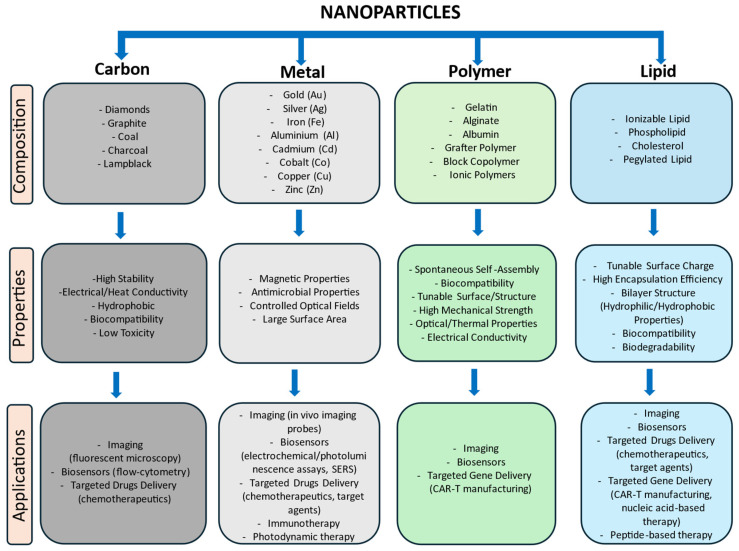
Schematic diagram of nanoparticles characteristics.

Since a large part of the human body is composed of carbon, an optimal strategy for the development of nanoparticles includes the use of biocompatible carbon-based materials [7]. Carbon nanoparticles (CNPs) are an extensive family of carbon allotropes that, according to their physical parameters (shape, size, and dimension), can be classified as zero-dimensional (carbon nanodots and carbon quantum dots), one-dimensional (carbon nanotubes and carbon nanofibers), two-dimensional (graphene), and three-dimensional nanomaterials (carbon sponges) [8,9,10,11,12]. CNPs have attracted wide attention due to their bioaffinities, durability, and lightweight properties, making them suitable for tissue engineering, biosensing, and bioimaging [13] (Figure 1). However, limitations such as high manufacturing costs have hampered the use of these nanostructures in clinical applications [14].

### 2.2. Metal Nanoparticles

Metal nanoparticles (MNPs) have raised a lot of interest in several biomedical fields due to their inert nature and nanoscale structures, which are similar in size to many biological molecules. A variety of MNPs have been developed using different metal elements and tested for various applications [15]. 

Gold nanoparticles (AuNPs) have been studied for diagnostic and therapeutic purposes because of their high X-ray absorption coefficient, ease of synthesis and manipulation, and distinct electronic properties. The negative charge on the surface of AuNPs allows their functionalization with organic molecules such as ligands, antibodies, or drugs, making them suitable for drug delivery [16]. 

Silver nanoparticles (AgNPs) applications have also significantly grown in biomedicine in the last few years. AgNPs can efficiently transfer electrons and have demonstrated high stability in water. AgNPs slowly release silver ions at low concentrations, providing intrinsic antimicrobial and anticancer properties [17]. 

Copper and aluminum are among the most abundant metals in nature and their physical and chemical properties, together with their inexpensive costs, have attracted attention for medical purposes. Copper and aluminum NPs are currently being studied for various applications, such as bio- and electrochemical sensors [18]. 

Zinc is an essential component for human physiology and homeostasis, playing a crucial role in many enzymes. The high biocompatibility, biodegradability, and low toxicity of zinc oxide NPs make them an attractive tool for several biomedical applications, including drug delivery and imaging [19]. 

Other types of MNPs, such as platinum, titanium, and iron NPs, have also attracted the interest of the biotechnological and pharmacological industries [20]. 

MNPs are particularly advantageous in cancer treatment due to their precise control over their shape, size, charge, and surface modification. Moreover, they are more easily taken up by cells compared to non-metallic NPs of the same size, providing a distinct advantage for cancer therapy [15] (Figure 1).

### 2.3. Polymeric Nanoparticles

Natural and synthetic polymers are among the most promising materials for NPs production, ensuring essential requirements for their application in clinical settings. These requirements include biocompatibility, bioavailability, biodegradability, and non-immunogenicity [21]. The great versatility of polymeric nanoparticles (PNPs) allows them to customize their structures based on the final applications for which they are designed. PNPs can be manufactured through chemical modifications directly on natural biopolymers or synthetically from natural monomers, leading to the generation of many nanostructures suitable for a wide range of applications [22]. Generally, polysaccharides and proteins such as alginic acid, gelatin, polylactic acid, chitosan, polylactide-co-glycolide, and polycaprolactone are used as precursors for PNPs fabrication [23]. PNPs are categorized into two forms:nanospheres and nanocapsules. In nanospheres, the bioactive molecules are integrated into a polymeric matrix, whereas in nanocapsules, the compounds are covered by polymers in the nanostructure core. These NPs allow the encapsulation and retention of high concentrations of active molecules, including volatile compounds [24]. The advantages offered by PNPs mainly involve controlled drug release, making them suitable for gene therapy or drug delivery to specific tissues or organs. Additionally, PNPs are used as vehicles for vaccines [25] (Figure 1).

### 2.4. Lipid Nanoparticles

Lipid nanoparticles (LNPs) have shown remarkable pharmacological and therapeutic properties, raising the interest of researchers for preclinical and clinical studies. Since the materials used for LNPs production include natural sources, they offer several benefits over other materials. These advantages include temporal and thermal stability, ease of preparation, high loading capacity, relatively low production costs, and feasibility for large-scale industrial manufacturing [26]. The main natural sources used for LNPs production include (i) phospholipids and cholesterol, which enhance the stability and circulation capability of NPs within the biological matrix, and (ii) positively charged lipids, which improve the loading capacity of negatively charged molecules. Additionally, LNPs chemical modifications, such as conjugation with gangliosides or polyethylene glycol (PEG), increase solubility and prevent detection by the immune system [27]. LNPs efficiently penetrate cell membranes and deliver their cargo to intracellular target sites due to their similarity to the lipid components of the biological membranes [28]. Once internalized, LNPs release the encapsulated bioactive molecules by exploiting the low pH of the intracellular environment. LNPs can also be linked to antibodies to recognize malignant cells or receptors [26] (Figure 1). 

The LNPs most commonly used in the biomedical field can be categorized into three main groups: liposomes, solid lipid nanoparticles (SLN), and nanostructured lipid carriers (NLC) [29]. Liposomes are composed of amphipathic phospholipids organized in bilayer structures. In aqueous solutions, liposomes form vesicles that enhance the stability and solubility of the cargo molecules, whether hydrophilic or hydrophobic. The permeability of hydrophobic drugs across the liposomal membrane can also be improved by adding cholesterol to the formulation. SLNs are composed of mono-, di-, or triglycerides, fatty acids, and glycerides. These physiological elements maintain a solid state in the body, ensuring physical stability over long periods, controlled release of both lipophilic and hydrophilic drugs, and protection of labile molecules. Furthermore, SLNs exhibit low or no toxicity, easy preparation, and low synthesis costs. Despite this, SLNs present limitations in terms of the loading and releasing capacity of cargo molecules [30]. These limitations are addressed by NLC, which is composed of a mixture of solid and liquid lipids such as glyceryl tricaprylate, ethyl oleate, isopropyl myristate, and glyceryl dioleate. These nanostructures represent an evolution of SLNs, capable of loading higher amounts of hydrophilic or hydrophobic molecules. NLC also exhibits high specificity against target sites due to the possibility of modifying their surface composition, thus increasing control over drug release and reducing toxicity [29]. LNPs-based therapy is currently applied in cancer treatment and other diseases, including the COVID-19 vaccine [31] (Figure 1).

## 3. Use of Nanoparticles in the Diagnosis and Monitoring of Lymphoid Malignancies

### 3.1. Lymphoid Leukemias

The diagnosis and classification of leukemia subtypes rely on the integrative analysis of morphological parameters, immunohistochemistry, flow cytometry, fluorescence in situ hybridization, PCR-based molecular testing, and DNA sequencing [4]. Continuous advances in biotechnologies have improved sensitivity, but the detection of leukemic cells in very low numbers remains a challenge for monitoring drug responses. In this context, NPs have been proposed as a promising tool for detecting neoplastic cells in various leukemia subtypes. Diagnostic techniques based on nanotechnology are being developed to detect cancer cells through the recognition of specific biomarkers. The large surface area-to-volume ratio of NPs allows for the conjugation of antibodies, aptamers, and other molecules on their surface that recognize specific cancer biomarkers [32,33,34]. In this section, we will focus on the application of NPs in diagnosing different subtypes of lymphoid neoplasms.

#### 3.1.1. Acute Lymphoblastic Leukemia

Acute lymphoblastic leukemia (ALL) arises from hematopoietic cells in either a B-(B-ALL) or T-(T-ALL) cell lineage precursor. ALL is genetically heterogeneous, with various and distinct genetic abnormalities associated with different subtypes [35,36]. In the recent past, NPs-based biosensors have been tested to detect specific ALL biomarkers, aiming to improve sensitivity and specificity for real-time monitoring of drug responses. 

In B-ALL, detecting the Philadelphia chromosome resulting from the BCR-ABL1 fusion gene has important implications for determining eligibility for targeted therapies with tyrosine kinase inhibitors (TKIs) Inizio moduloand and for monitoring MRD to implement treatment choices [37]. AuNPs coupled with DNA probes targeting the BCR/ABL fusion gene have been developed to allow the detection of the BCR/ABL oncogene with a detection limit of 1.0 pM in B-ALL samples. The specific hybridization between the NPs-probes and BCR/ABL targeted genes was demonstrated by changes in the amperometric current, which were linearly related to the concentrations of the DNA target in the tested samples [38]. A similar approach to detecting the BCR/ABL chimeric oncogene was performed using a polyaniline-gold composite. These electrochemical biosensors displayed high sensitivity and specificity and were able to detect up to 41 cDNA copies per μL in B-ALL samples [39]. The detection of the BCR/ABL fusion gene with these NPs-based molecular assays showed an enhanced detection limit as compared to the gold standard PCR assays and required fewer steps.

**Figure 2:**
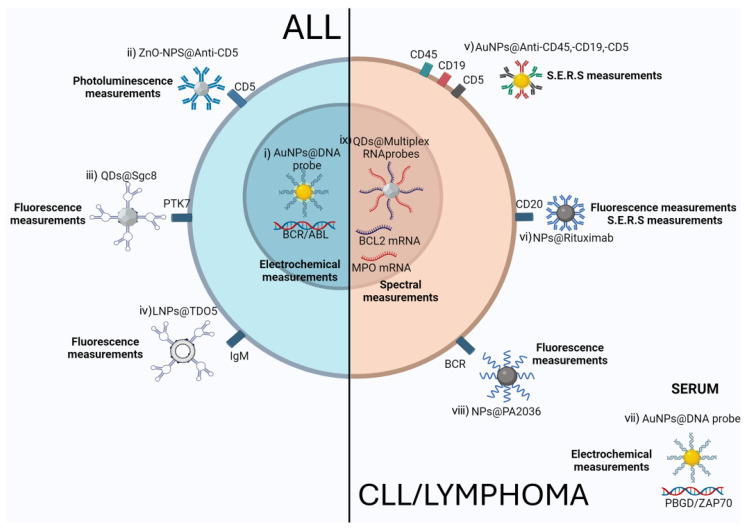
NPs diagnostic applications in lymphoid diseases. (i) Gold NPs (AuNPs) coupled with DNA probes targeting the BCR/ABL fusion gene. Detection performed through electrochemical measurements. (ii) Oxide Zinc NPs conjugated with anti-CD5 antibody (ZnO-NPs@Anti-CD5). Detection performed through photoluminescence measurements. (iii) Quantum Dots conjugated with Sgc8 aptamer (QDs@Sgc8) target the tyrosine kinase 7 protein (PTK7). Detection performed through fluorescence measurements. (iv) Lipid NPs conjugated with TDO5 aptamer (LNPs@TDO5) target immunoglobin heavy mu chain receptor. Detection performed through fluorescence measurements. (v) AuNPs conjugated with anti-CD45, anti-CD19, and anti-CD5 antibodies. Detection performed through S.E.R.S. measurements. (vi) NPs (polymeric, magnetic, quantum dots, albumin, silver) conjugated with anti-CD20 antibody Rituximab (NPs@Rituximab). Detection performed through fluorescence or S.E.R.S. measurements. (vii) AuNPs coupled with DNA probes targeting a specific mutated sequence of porphobilinogen deaminase gene (PBGD) or a specific sequence of ZAP70 gene in serum samples. Detection performed through electrochemical measurements. (viii) Diatomite NPs coupled with PA2036 peptide targeting BCR. Detection performed through fluorescence measurements. (ix) Quantum Dots conjugated with oligonucleotide probes (QDs@MultiplexRNAprobes) targeting BCL2 and MPO transcripts. Detection performed through spectral measurements.

NPs have also been used to recognize membrane-bound biomarkers expressed on ALL cells. Wu et al. developed a nanocomplex composed of the TDO5 aptamer with attached lipid tails through a PEG linker. The TDO5 aptamer is an oligonucleotide that forms noncovalent interactions with the immunoglobin heavy mu chain receptor, found overexpressed in a B-ALL cell line. These micelle nanostructures enabled rapid identification of leukemic cells within tested populations with low critical micelle concentrations [40]. Surface NPs biosensors were also tested for T-ALL detection. Specifically, zinc oxide nanostructures were used as a platform for conjugating monoclonal antibodies targeting the CD5 surface molecule and were tested as a photo-electrochemical immunosensor in T-ALL. The high selectivity of these NPs was demonstrated through photoluminescence assays. Photoluminescence intensity was correlated with the number of CD5-positive cells in the investigated populations even at extremely low cell concentrations, from 3 to 128 cells per milliliter [41]. Quantum dots (QDs) also showed promising results as a detection tool for ALL cells after functionalization with the Sgc8 aptamer that specifically recognizes the tyrosine kinase 7 protein (PTK7) expressed on a T-ALL cell line surface. This nanocomplex enabled tumor cell imaging in vitro and in vivo, providing the rationale for clinical application [42] (Figure 2, Table 1).

Overall, these promising nanotechnologies could represent a cost-effective and accurate detection tool for measuring ALL biomarkers, useful for monitoring MRD after therapy. 

#### 3.1.2. Chronic Lymphocytic Leukemia 

Chronic lymphocytic leukemia (CLL) is a B lymphoid neoplasm characterized by the monoclonal expansion of CD19+ CD5+ positive cells in blood, bone marrow, and lymphoid tissues. CLL diagnosis and monitoring rely on immunophenotyping, while the characterization of molecular biomarkers such as TP53 disruption, NOTCH1 mutation, immunoglobulin heavy chain variable region (IGHV) gene mutational status, and other genetic aberrations provides important parameters for CLL prognostication [43,44,45,46,47].

NPs have been explored to improve the accuracy of CLL immunophenotyping. MacLaughlin and colleagues combined the sensitivity and specificity of Raman spectroscopy with the unique physical and optical properties of AuNPs to develop PEG-coated surface-enhanced Raman scattering (SERS) AuNPs conjugated with anti-CD45, anti-CD19, and anti-CD5 antibodies simultaneously and demonstrated the high specificity of these nanotools in primary samples [48]. The proposed technology could further improve MRD sensitivity by exploiting the high resolution provided by SERS coupled with NPs. 

The evaluation of CLL leukemic burden has been explored in vivo using PNPs functionalized with the anti-CD20 chimeric antibody Rituximab and imaging agents. Capolla et al. showed the ability of these engineered NPs to detect tumor mass in CLL xenograft models by transplanting a CLL cell line in SCID mice. A diffuse distribution was observed throughout the bodies of the mice for up to 96 h, starting a few minutes after the intravenous injection of 1 nmol of Cy5.5-anti-CD20 NPs, providing insights into NP behavior in vivo as an imaging probe [49]. 

DNA-based NPs biosensors have also been investigated for the recognition of molecular biomarkers in CLL cells. A highly sensitive DNA sensor was constructed based on a porphobilinogen deaminase (PBGD) probe conjugated with AuNPs to detect specific mutated sequences of the PBGD gene, which is highly associated with CLL. Electrochemical impedance spectroscopic measurements demonstrated the high sensitivity of these nanosensors in detecting the complementary oligonucleotide sequences in CLL serum samples and discriminating between one-base mismatched, noncomplementary, and complementary oligonucleotide sequences. Furthermore, the nanosensors can be regenerated by removing the target DNA from the biological samples [50]. 

NPs have also been tested to predict CLL prognosis by engineering AuNPs with an oligonucleotide targeting a specific sequence of the ZAP70 gene associated with the IGHV mutational status. These nanocomplexes displayed high selectivity in detecting ZAP70 point mutations in primary CLL samples, with a detection limit of 4.0  ×  10^−15^ molL^−1^ [51]. This approach could be useful for fast screening of the IGHV mutational status (Figure 2, Table 1).

### 3.2. Lymphomas

Lymphomas comprise a heterogeneous group of lymphatic system neoplasms arising from the clonal proliferation of B or T lymphocytes at different stages of maturation. Based on cell morphology and the presence of Reed-Sternberg cells, lymphomas can be classified as Hodgkin (HL) or non-Hodgkin lymphoma (NHL). Diagnosing these malignancies involves invasive procedures such as excisional biopsy to evaluate the architecture of diseased lymph nodes. Other techniques based on immunophenotypical and molecular characterization improve diagnostic accuracy by providing additional parameters useful for the additional and complex sub-classification within the main lymphoma groups. However, many technical hurdles, mainly related to inadequate tissue sampling and the complexities in lymphoma classification (more than 70 species, according to the WHO classification), make diagnosis challenging [52]. Furthermore, biopsies may have limitations in capturing the full heterogeneity and dynamic nature of the tumor. In recent years, nanotechnologies have received considerable attention for addressing these diagnostic challenges. Detecting tumor cells from complex biological fluids could overcome the invasive procedures relied upon for lymphoma diagnosis, thus improving accuracy, sensitivity, and specificity. 

Targeting CD20 through functionalized NPs is a widely explored strategy to enhance the detection of B lymphoma cells. CD20 is overexpressed in these B-cell malignancies, and expression levels appear to correlate with the growth rate of lymphoma cells. A system composed of avidin-labeled magnetic NPs and biotin-labeled anti-CD20 antibodies was developed to specifically target and isolate a CD20-expressing lymphoma cell line. These NPs were tested in magnetic separation experiments against samples containing a mixture of CD20 positive and negative cell types, and the receptor recognition ability was found to be over 95%, highlighting the ease, time-saving, and cost-effective advantages of these nanotools [53]. The diagnostic value of nanoprobes has also been explored using fluorescent semiconductor QDs coupled with the rituximab monoclonal anti-CD20 antibody and tested against lymphoma cells and tissue samples collected from patients with Diffuse Large B-cell Lymphoma (DLBCL) using flow-cytometry and fluorescent microscopy, respectively. To confirm that this nanotool specifically binds cells overexpressing the CD20 biomarker, CD20-negative cells and tissue samples from healthy donors were used as negative controls. The sensitivity and specificity of the test were 100% and 89.5%, respectively, with positive and negative predictive values of 91.3% and 100%, respectively [54]. The low rate of false positives and false negatives demonstrated by this nanotechnology could provide accurate molecular characterization with high sensitivity, which is essential for the timely diagnosis and treatment of DLBCL. 

Imaging of the tumor burden has been explored in lymphoma xenograft models by labeling albumin-based NPs coated with rituximab and the Alexa Fluor 750 fluorochrome [55]. Alternatively, Vo-Dhin et al. exploited the excellent SERS properties of AgNPs and the specificity of the CD20 antibody Rituxan to discriminate lymphoma cells among mixed cell samples. The authors demonstrated the high sensitivity of this approach in the detection of diseased living cells at the single-cell level and highlighted the importance of nanoprobe size, showing that the Raman signal intensity of 50 nanometers (nm) NPs was 10 fold higher compared to 10 nm NPs, indicating a particle-size-dependent effect. This evidence suggests that optimizing NPs size [56] could regulate the sensitivity and specificity of NPs in detecting cancer cells at the single-cell level. 

Improving the sensitivity and specificity of detection tools could be relevant to providing important insights into the heterogeneous nature of circulating tumor cells captured from peripheral blood samples and even cancer tissue biopsy [57]. A specific tumor antigen expressed by lymphoma cells could be represented by the hypervariable region of the surface immunoglobulin B-cell receptor (BCR), which is unique to each clonal B-cell population. Martucci and colleagues generated FITC-labeled inorganic diatomite NPs conjugated with the pA2036 peptide that specifically binds the Ig-BCR expressed on the murine A20 lymphoma cell line. The authors showed that the detection and uptake of this nanocomplex in A20 cells were threefold higher compared to other surface IgG-positive B-cell lines unable to bind the pA2036 peptide [58]. 

Useful approaches to predicting the clinical outcomes of lymphoma patients include gene expression profiling and multiplatform genetic approaches that distinguish genotypic and epigenic clusters. In the context of DLBCL, gene expression profiling has helped uncover two main subtypes: Activated B Cell (ABC) and Germinal Center B cell (GCB) DLBCL. Other genotypic and epigenetic stratifications of DLBCL subtypes rely on the evaluation of BCL-6 and NOTCH2 mutations (referred to as BN2 subtypes) or NOTCH1 mutations (N1 subtypes) [59]. However, the high-quality tissue samples required for these analyses may not always be available in clinical practice. Tholouli et al. developed a methodology for multiplexed in situ hybridization using QDs coated with oligonucleotide probes on formalin-fixed paraffin-embedded (FFPE) human biopsies. This nanoprobe enabled the simultaneous and quantitative detection of MPO and BCL2 transcripts in bone marrow FFPE infiltrated by follicular lymphoma cells through spectral imaging analysis [60]. This approach could facilitate the translation of gene expression profiling into clinical practice, significantly impacting lymphoma prognosis and treatment efficacy (Figure 2, Table 1).

**Table 1:** Summary of selected studies using NPs for the detection of lymphoid cancer cells.

Type of NPs	Nanomaterial	Functionalization	Tumor	Target	Detection Method	Advantages
Carbon	Diatomite	pA2036 peptide	Murine B cell lymphoma	Ig-BCR	Flow-cytometry, fluorescent microscopy	High specific internalization efficiency and lack of cytotoxicity [58]
Metal	Gold	DNA probe	B-ALL	BCR/ABL	Amperometry	Enhanced detection limit as compared to the gold standard PCR assays [38]
		Monoclonal Abs	CLL	CD45-CD19-CD5	SERS	Enhanced sensitivity as compared to flowcytometry gold standard assay [48]
		DNA probe	CLL	PBGD	Electrochemical measurements	High sensitivity allowing to discriminate one-base mismatched, noncomplementary and complementary oligonucleotides sequence [50]
	Cadmium and selenium QDs	Monoclonal Abs	DLBCL	CD20	Flow-cytometry, fluorescent microscopy	Low rate of false-positive and false-negative [54]
		Oligonucleotide probes	Follicular lymphoma	MPO-BCL2	Spectral imaging analysis	Simultaneous and quantitative detection of MPO and BCL2 transcripts on FFPE specimens [60]
	Oxide Zinc	Monoclonal Abs	T-ALL	CD5	Photoluminescence detection	High selectivity and specificity at extremely low cell concentrations [41]
	Silver	Monoclonal Abs	Burkitt lymphoma	CD20	SERS	High sensitivity in the detection of diseased living cells at single-cell level [56]
Lipid	Lipid tail with diacyl chains	TDO5 aptamer	B-ALL	Ig heavy mu chain receptor	Flow cytometry, fluorescent microscopy	Rapid identification of leukemic cells with low critical NPs concentration [40]
Polymeric	Carboxylic acid-terminated polymers	Monoclonal Abs	CLL	CD20	Flow cytometry, NIR fluorescence-imaging	In vivo tumor mass evaluable within 24 h after intravenous injection [49]

## 4. Nanoparticles in the Treatment of Lymphoid Malignancies

### 4.1. Lymphoid Leukemias

#### 4.1.1. Acute Lymphoblastic Leukemia

The treatment of ALL has been revolutionized by the introduction of TKIs, which have drastically improved patient outcomes. However, the clonal heterogeneity of ALL can result in only partial elimination of leukemia cells upon targeted therapy, allowing resistant clones to be selected and leading to relapse with a poor clinical outcome [61].

##### NPs as Carriers for Tyrosine Kinase Inhibitors

**Figure 3:**
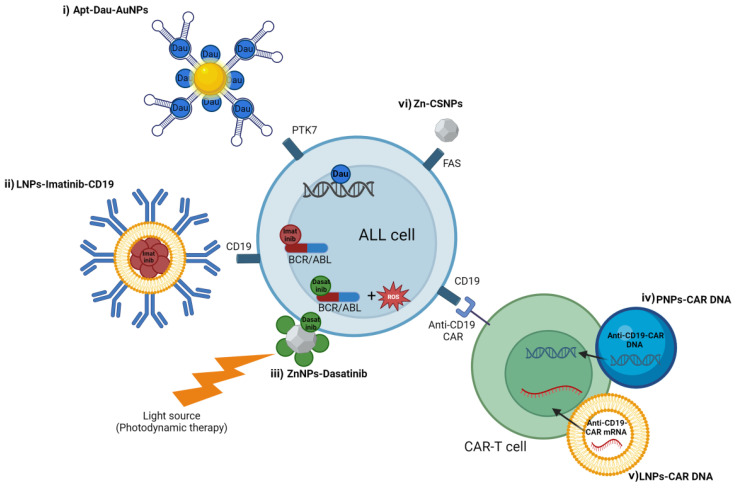
NPs applications in ALL treatment. (i) Gold NPs conjugated with Sgc8 aptamer targeting PTK7 and daunorubicin (Apt-Dau-AuNPs). Daunorubicin interacts with DNA to inhibit macromolecular biosynthesis. (ii) Lipid NPs conjugated with anti-CD19 antibody and the BCR/ABL inhibitor imatinib. Imatinib inhibits the BCR-ABL protein by binding to the ATP pocket in the active site, preventing downstream phosphorylation of target protein. (iii) Zinc(II) phthalocyanine conjugated with dasatinib (ZnNPs-Dasatinib). ZnNPs act as a photosensitizer able to generate reactive oxygen species (ROS) after stimulation with a light source at specific wavelength, while dasatinib works by binding close to the ATP binding site of the BCR-ABL protein. (iv) Polymeric NPs carrying anti-CD19 CAR DNA (PNPs-CAR DNA) and (v) lipid NPs carrying anti-CD19 CAR mRNA (LNPs-CAR mRNA) for the delivery of leukemia-targeting CAR genes into the nuclei of circulating T cells. (vi) Chitosan polymeric NPS containing Zn (Zn-CSNPs) triggers the activation of Fas receptor with the consequent caspase-cascade activation.

Liposome-based NPs have been proposed to improve the efficacy of TKIs in B-ALL treatments. An immunoliposome carrying an anti-CD19 antibody has been developed to deliver the BCR/ABL inhibitor imatinib specifically to Philadelphia chromosome-positive (Ph+) ALL cells. The high specificity and absorption efficacy of PEG-liposomes coated with the anti-CD19 monoclonal antibody rely on the high expression of CD19 in B-cell lineage cells, along with the rapid internalization of CD19 after antibody binding. Harata et al. demonstrated the specificity of this nanotechnology through a competition assay using free CD19 antibodies. The internalization of the nanostructures in ALL cell lines was inhibited by CD19 antibodies in a dose-dependent manner. The authors also demonstrated the higher apoptotic effects of these nanodrugs in Ph+ ALL cell lines and primary cells compared to free imatinib, while Ph-negative cells were resistant to the NPs cytotoxic effects. This strategy showed the potential to reduce the off-target and off-tumor side effects of free imatinib. Additionally, these immunoliposomes were also effective in imatinib-resistant cells from ALL patients, suggesting that these nanotherapeutics could improve responses in relapsed cases [62]. Other studies provided promising therapeutic strategies for T-ALL by combining zinc (II) phthalocyanine with dasatinib, resulting in enhanced targeting, immunomodulation, and cytotoxicity against leukemic T cells. In animal studies, this nanocomplex demonstrated a rapid elimination rate of ALL cells from the body and exhibited remarkable tumor regression compared to free dasatinib, even at nanomolar concentrations, indicating its potential as an effective therapeutic agent for ALL [63] (Figure 3, Table 2). These nanoformulations demonstrate that they enhance the antitumor effects of TKIs with higher specificity and reduced side effects, and they also induce strong immune responses as immunomodulators, potentially leading to secondary destruction of cancer cells and systemic anticancer immunity.

##### NPs as Carriers for Chemotherapeutic Agents

A complex delivery system has been proposed to specifically deliver and internalize daunorubicin into T-ALL cells. This nanotechnology consists of AuNPs functionalized for the specific recognition of T-ALL cells using the sgc8c aptamer (Apt-Dau-AuNPs), which targets PTK7. The study demonstrated that daunorubicin can be adsorbed onto this nanosystem due to the electrostatic interaction between the positive charge of daunorubicin and the negative charge of AuNPs. Confocal images showed the internalization of FAM-labeled nanocomplexes into the Molt-4 T-ALL cell line, which was associated with a drastic reduction in cell viability. A pH value of 5.5, resembling intracellular endosomes, lysosomes, or cancerous tissue conditions, enhanced the rate of daunorubicin release, potentially improving treatment efficiency with a lower amount of drug, reduced side-effects, less frequent administration, and increased patient convenience and compliance [64]. A drug delivery strategy targeting T-ALL cells with doxorubicin was developed using a metabolic reprogramming L-phenylalanine polymer. In vivo drug distribution experiments showed that T-ALL cells have enhanced cellular uptake of L-phenylalanine compared to normal hematopoietic cells. The L-phenylalanine polymer assembled with doxorubicin specifically targeted T-ALL cells while sparing normal hematopoietic cells, with enhanced in vivo anti-leukemic efficacy and reduced side effects. Additionally, this nanocomplex promoted the activation of immune surveillance by inhibiting glucose metabolism in myeloid-derived suppressor cells (MDSCs) by targeting pyruvate kinase 2 (PKM2) [65] (Figure 3, Table 2).

##### NPs for Chimeric Antigen Receptor T Manufacturing

Chimeric antigen receptor (CAR) T cells targeting surface receptors have shown significant anti-leukemic effects in ALL. To date, CAR-T manufacturing is a complex process involving several key in vitro steps for genetic modification with retroviral or lentiviral vectors. Nanotechnology presents an intriguing opportunity to simplify this process by delivering genes encoding disease-specific CARs into patient-derived T cells. NPs have been explored to enhance this expensive and time-consuming protocol through polymeric nanocarriers that facilitate the in situ delivery of leukemia-specific CAR genes into circulating T cells. A study showed that DNA-carrying NPs could efficiently deliver leukemia-targeting CAR genes into the nuclei of T cells in ALL mouse models, resulting in long-term disease remission and highlighting the potential effectiveness of this approach in treating ALL [66]. NPs could also offer a potential solution to mitigate the adverse effects associated with viral vectors commonly used for delivering CAR genes. NPs composed of ionizable lipids have been employed to encapsulate CAR mRNA, enabling the generation of functional CAR T cells. This approach allows for transient expression of CAR without genomic integration. In the context of B cell malignancies, CD19-directed CAR T cells target both cancerous and normal B cells, which can lead to the elimination of both neoplastic and normal CD19-positive cells, resulting in B cell aplasia and hypogammaglobulinemia. Compared to viral and electroporation-based delivery systems, LNPs induce lower levels of T cell toxicity while achieving similar levels of CAR surface expression. LNP-engineered CAR T cells exhibited comparable ALL cell-killing efficacy to both electroporation- and virally-engineered CAR T cells [67] (Figure 3, Table 2).

##### NPs as Anti-Leukemic Agents

NPs have also exhibited intrinsic anti-leukemic properties related to the unique characteristics of their constituent materials. Chitosan (CS) polymeric NPs containing Zn (Zn-CSNPs) have been developed for treating ALL by exploiting the apoptotic properties of Zn against leukemic cells. Zn-CSNPs have been demonstrated to reduce ALL cell growth by triggering the activation of the Fas receptor with the consequent caspase-cascade activation and secretion of apoptotic cytokines. Zn-CSNPs also showed the ability to release Zn slowly at the normal or ALL blood cell pH cell, thereby reducing the risk of overdose and preventing cellular damage [68] (Figure 3, Table 2).

#### 4.1.2. Chronic Lymphocytic Leukemia (CLL)

Advances in CLL treatment have significantly improved patient outcomes over the years. Targeted agents used in CLL specifically attack cancer cells by interfering with the BTK and BCL2 pathways, which are crucial for cell growth and survival. Preclinical studies have also identified other molecular targets, such as NOTCH1, whose inhibition has shown antileukemic effects both in vitro and in vivo models of CLL [69,70,71,72]. Despite these advances, treatment resistance in CLL can still arise due to intrinsic factors, acquired mutations, microenvironmental protection, clonal evolution, bypass pathways, immune evasion, and stromal interactions. Additionally, these drugs can cause various severe side effects, which may differ depending on the specific treatment regimen and individual patient factors [73].

NPs-based treatments for CLL have demonstrated enhanced therapeutic efficacy through several mechanisms, including targeted drug delivery, immunomodulation, and sustained drug release. NPs also enable the co-delivery of multiple therapeutic agents, allowing for synergistic effects and the overcoming of resistance mechanisms in CLL. Additionally, NPs can overcome biological barriers, such as the protective microenvironment within lymphoid tissues, enabling drugs to reach CLL cells in difficult-to-access locations.

##### Use of NPs in Immunotherapy

**Figure 4:**
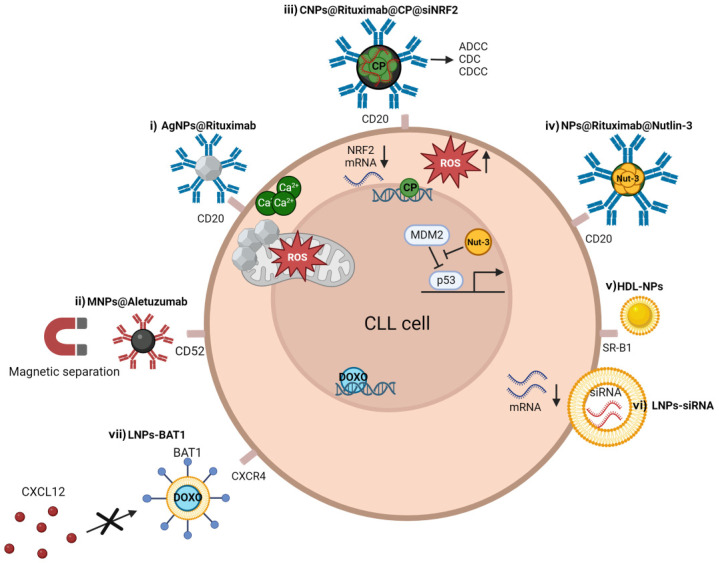
NPs applications in CLL treatment. (i) Silver NPs conjugated with the anti-CD20 antibody rituximab (AgNPs@Rituximab). AgNPs lead to mitochondrial-driven apoptosis, triggered by increased calcium influx and ROS overproduction. (ii) Magnetic NPs conjugated with the anti-CD52 antibody aletuzumab (MNPs@Aletuzumab). Magnetic columns allow for the removal of CLL cells. (iii) Chitosan (CS) polymeric NPs conjugated with the monoclonal antibody rituximab and loaded with the chemotherapeutic drug cyclophosphamide and an anti-NRF2 siRNA (CNPs@Rituximab@CP@siNRF2). Rituximab allows CD20 targeting and induces antibody-dependent cellular cytotoxicity (ADCC), complement-dependent cytotoxicity (CDC), and complement-dependent cell-mediated cytotoxicity (CDCC). Cyclophosphamide works by interfering with the duplication of DNA, while siNRF2 induces ROS accumulation. (iv) NPs engineered with rituximab and the nutlin-3 inhibitor. Nutlin-3 inhibits the interactions between MDM2 and p53. (v) Synthetic AuNPs-high-density lipoprotein NPs (HDL-NPs) binding SR-B1. HDL-NPs affect the cholesterol metabolism in CLL cells. (vi) Lipid-based NPs loaded with small interfering RNA (LNPs-siRNA). LNPs allow NPs to efficiently penetrate cellular membranes, maximizing siRNA stability and cellular uptake. (vii) Lipid NPs conjugated with BAT1 and loaded with doxorubicin (LNPs-BAT1-DOXO). BAT-1 binds CXCR-4 to antagonize intracellular signal generation, cell migration, and prosurvival effects of CXCL12. Doxorubicin intercalates into DNA and disrupts topoisomerase-II-mediated DNA repair.

A strategy proposed to improve CLL immunotherapy involves conjugating monoclonal antibodies to the surface of NPs to specifically target and eliminate cancer cells. Several preclinical studies have shown that the specificity of monoclonal antibodies can enhance the intrinsic anti-leukemic properties of nanomaterials. We developed AgNPs conjugated with rituximab and demonstrated the specific internalization of these nanocomplexes into CLL cells even after 0.5 h of treatment. The anti-leukemic effects of rituximab-coupled AgNPs were highlighted in in vivo experiments, where the survival rates of CLL xenograft models treated with this nanocomplex were significantly increased compared to mice treated with vehicle or unconjugated single agents. This study also demonstrated the synergistic effects of AgNPs in combination with ibrutinib or venetoclax, which could be exploited for combination therapies [74]. Alemtuzumab-coupled NPs have also been tested to specifically target and remove circulating CLL cells from blood patients. This approach was feasible due to the magnetic properties of carbon-coated cobalt NPs used for the conjugation with alemtuzumab. The resulting nanoconjugates, when added to blood samples from CLL patients, were isolated using magnetic columns, allowing for the removal of about 50% of neoplastic cells. A second purification step or higher concentration of NPs further improved the removal of CLL cells [75] (Figure 4, Table 2).

##### NPs as a Drug Delivery Platform

The use of NPs as a multiple-drug delivery platforms has been proposed to reduce systemic toxicity and enhance drug efficacy in CLL. Chitosan (CS) polymeric NPs, conjugated with the monoclonal antibody rituximab and loaded with the chemotherapeutic drug cyclophosphamide and an anti-NRF2 siRNA, were generated to target CLL cells through different mechanisms. NRF2 is a transcription factor overexpressed in CLL cells, playing a critical role in cellular defense against oxidative stress and inflammation, and its deregulation has been implicated in cancer drug resistance. This nanocomplex demonstrated effective uptake in primary CLL cells, exerting significant anti-leukemic activity at a lower concentration compared to free cyclophosphamide. Genetic silencing of NRF2 further increased the sensitivity of CLL cells to cyclophosphamide, suggesting a synergistic role of anti-NRF2 siRNA and cyclophosphamide [76]. The use of NPs as vehicles for multiple-drug delivery could overcome the potential increased toxicity and side effects. Combining multiple drugs can lead to additive or synergistic toxicities, which may limit treatment adherence. The use of NPs as a multiple-drug delivery platform showed the potential to reduce systemic toxicity and enhance drug efficacy in CLL. The potential of NPs as drug delivery platforms was also explored and validated in other hematological diseases. For instance, in acute promyelocytic leukemia, ferritin-based NPs were employed to improve the delivery of trivalent arsenic (As^III^) to leukemic cells. This technology levered the high affinity of ferritin for CD71, which is strongly expressed by leukemic cells, demonstrating its potential as a precision treatment with low effective concentrations of As^III^ [77] (Figure 4, Table 2).

##### NPs and p53 Targeting

NPs engineered with rituximab have also been utilized in CLL to deliver the nutlin-3 inhibitor specifically to CD20+ target cells, aiming to interfere with the p53/MDM2 axis. Nutlin-3 has demonstrated anticancer activity in various p53 wild-type cancers, but its clinical applications have been limited by nonspecific targeting. This nanotool was shown to specifically induce cell death in CLL cells in vitro and to promote activation of the complement cascade in the presence of human serum. Promising therapeutic activity was also observed in CLL xenograft models, where it significantly improved survival rates compared to control animals [78] (Figure 4, Table 2).

##### NPs Targeting Cholesterol Metabolism

Modulation of mitochondrial metabolism and BCR signaling in CLL suggests that targeting cell metabolism represents a promising therapeutic approach in CLL. CLL cells exhibit distinct metabolic alterations compared to normal lymphocytes, including increased glucose uptake, mitochondrial dysfunction, redox imbalance, and altered lipid metabolism. In particular, cholesterol metabolism is emerging as a new potential target in CLL. The scavenger receptor type B-1 (SR-B1), a high-affinity receptor for cholesterol-rich high-density lipoprotein (HDL), has been shown to be selectively expressed in CLL cells compared to healthy lymphoid cells. McMahon et al. suggested that SR-B1 could be an effective molecular target to affect the cholesterol metabolism in CLL cells, offering therapeutic benefits. For this purpose, synthetic HDL NPs were generated starting from a 5 nm-diameter AuNP as a precursor and tested against CLL cells to target SR-B1. The data showed that HDL NPs specifically induced cell death in CLL cells while sparing the viability of normal B and T cells isolated from healthy volunteers [79] (Figure 4, Table 2).

##### NPs and Nucleic Acid-Based Therapy

Lipid-based NPs have also been tested as delivery vehicles for mRNA or siRNA to inhibit the expression of specific genes associated with cancer. Current limitations that hinder the clinical translation of RNA-based therapies in CLL include RNA instability, poor cellular uptake, toxicity, and short in vivo circulation times. Biocompatible lipid-based NPs formulations can encapsulate nucleic acids, maximizing RNA stability and cellular uptake while reducing the risk of adverse reactions or immune responses. In CLL, the rapid uptake kinetics of LNPs encapsulating Cy5-labeled control siRNA or luciferase-coding mRNA were evaluated using flow cytometry and confocal microscopy imaging, demonstrating that the rapid uptake of LNPs is a key factor in improving RNA transfection efficacy in cancer cells. The co-administration of RNA-LNPs with non-toxic concentrations of resveratrol to CLL cells further enhanced the cellular uptake of both mRNA- and siRNA-LNPs [80] (Figure 4, Table 2).

##### Use of NPs to Inhibit Microenvironmental Interactions

Delivery of small-molecule inhibitors using LNPs has been investigated in CLL to target the CXC chemokine receptor 4 (CXCR4), found to be overexpressed in CLL cells. CXCR4 plays a significant role in the interaction between neoplastic cells and their microenvironment through binding with CXC chemokine ligand 12 (CXCL12). The CXCR4–targeting drug BAT1 was incorporated into liposomes, and its binding affinity to CXCR4 on primary CLL cells was demonstrated using flow cytometry and antibody competition assays. BAT1-LNPs were loaded with a high amount of doxorubicin, and flow-cytometry analysis showed that this nanoformulation induced cell death after 24 h of treatment, suggesting the potential to deliver an increased local dose of therapeutics and disrupt cancer cells interaction with their chemoprotective niche [81] (Figure 4, Table 2). Targeting the interaction between ligands and cell receptors to block or modify key cell functions, such as intracellular signal generation, cell migration, and prosurvival stimuli, could be an effective strategy to achieve therapeutic effects and chemosensitize cancer cells.

### 4.2. Lymphoma

Currently, lymphoma treatment primarily relies on standard chemotherapy and immunotherapeutic approaches. In recent decades, these strategies have been implemented with innovative and effective advances, including targeting agents, CAR-T cells, and bispecific antibodies that showed improved outcomes. However, resistance and relapse can occur due to the complex and heterogeneous mutational landscape of lymphomas. Advances in biomarker testing and genetic profiling have been crucial in identifying novel determinants with targetable potential, guiding treatment strategies, and reducing the risk of relapse.

#### 4.2.1. NPs as Carriers for Chemotherapeutic Agents

NPs have been investigated as a potential treatment approach for lymphoma, showing promising results in preclinical studies. A key advantage of using nanotechnology in lymphoma treatment is the potential to deliver high doses of anticancer drugs specifically to cancerous cells within hard-to-reach areas, such as lymph nodes, while sparing healthy tissue. LNPs engineered with the topoisomerase I poison SN-38 have been developed to deliver effective amounts of the chemotherapeutic agent to tumor sites in lymphoma mouse models using polyclonal T cells as live nanocarriers. These cells were coated with NPs after stimulation aimed to confer SN-38 resistance and expression of lymphoid tissue homing receptors. After injection into mice, NPs-decorated T cells were found dispersed throughout the lymph node, in close proximity to cancer cells. SN-38 accumulation was significantly increased in tumor-bearing lymph nodes and remained elevated for at least 4 days [82]. Other innovative strategies based on the homing capability of live nanocarriers to deliver anticancer molecules in tumor niches have been successfully demonstrated in myeloid tumors, with a potential translational impact for lymphoid diseases. In acute myeloid leukemia (AML), a delivery system obtained by the cellular combination of hematopoietic stem cells (HSC) and platelets facilitated the transport of anti-PD-1 antibodies covalently conjugated to the surface of platelets in the bone marrow niches of AML in vivo models. The activation of platelets generated platelet-derived microparticles (PMPs) that released the anti-PD-1 antibody, leading to a T-cell immune response capable of inhibiting leukemia growth [83]. Similarly, liquid nitrogen-based cryo-shocked AML cells were employed as live drug carriers to deliver doxorubicin in the bone marrow of AML in vivo models. These cryo-shocked AML cells maintained intact cell structures, allowing for doxorubicin encapsulation, but lost their proliferative ability and pathogenicity. These cells also retained their bone marrow homing capability and demonstrated the ability to stimulate an immune response, which cooperated with doxorubicin to eradicate leukemia in tumor-bearing mice [84]. Alternatively, a biomimetic, bone marrow-specific targeted nanomedicine was generated using MnO2 NPs loaded with doxorubicin and camouflaged in the plasma membrane of an AML cell line [85].

A strategy to improve the efficacy and safety of chemotherapeutic agents in lymphoma treatment was developed using Fe_3_O_4_@SiO_2_- MNPs conjugated with cytarabine. Magnetically guided drugs involve an external magnetic field to deliver nanoparticles to a desired target area where the medication is needed. Cytarabine-MNPs were tested on lymphoma cell lines, and DNA binding studies showed that DNA aggregated on cytarabine-MNPs via groove binding, and the in vitro cytotoxic activity of cytarabine-MNPs was found to be two orders of magnitude higher than that of free cytarabine [86]. The advantage of this nanocomplex, when coupled with an extracorporeal magnetic device, is that it allows for reduced drug dosage and minimizes the systemic effect, thereby localizing the anticancer molecules to the targeted area.

**Figure 5:**
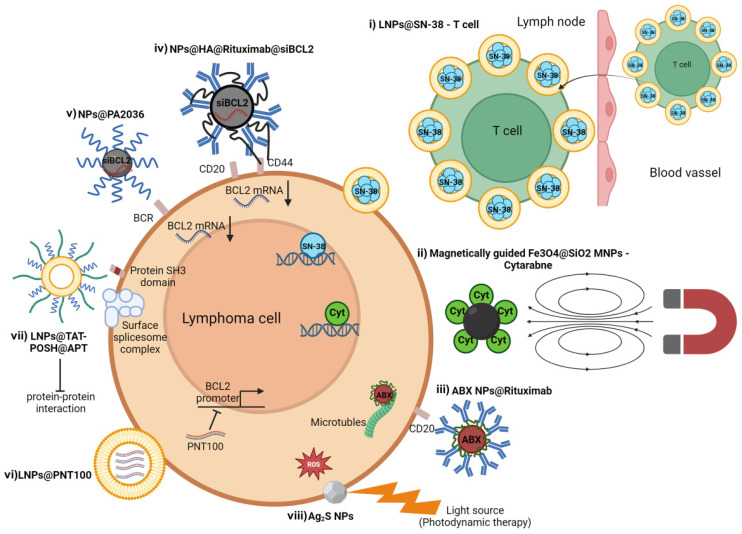
NPs applications in lymphoma treatment. (i) LNPs engineered with topoisomerase I poison SN-38 (LNPs@SN-38) loaded on T cells used as live nanocarriers to deliver anticancer drugs to cancerous cells within hard-to-reach areas. SN-38 binds to DNA, leading to the formation of lethal DNA breaks. (ii) Fe_3_O_4_@SiO_2_-magnetic nanoparticles conjugated with cytarabine (Fe_3_O_4_@SiO_2_ MNPs–Cytarabine). Fe_3_O_4_@SiO_2_ MNPs–Cytarabine involves an external magnetic field to deliver nanoparticles to a desired target area. Cytarabine interferes with DNA replication. (iii) NPs composed of nab-paclitaxel (ABX) and the anti-CD20 antibody rituximab (AR160). AR160 affects microtubule function. (iv) NPs engineered with polysaccharide hyaluronic acid (HA), anti-CD20 antibody rituximab, and siRNA targeting the BCL-2 oncogene. The dual binding of HA and rituximab with CD44 and CD20, respectively, improves NPs cellular uptake. (v) diatomite NPs containing anti-BCL2 siRNA and engineered with an idiotype-specific peptide targeting the BCR (NPs@PA2036). (vi) LNPs encapsulating PNT100 (LNPs@PNT100). PNT100 binds the regulatory region upstream of the BCL-2 gene to inhibit its transcription. (vii) LNPs coated with the therapeutic peptide Tat-POSH and a lymphoma-specific aptamer (LNPs@TAT-POSH@APT). Aptamer targets a ribonucleotide surface complex on the surface of lymphoma cells, while Tat-POSH targets the protein SH3 domains to inhibit protein-protein interaction. (viii) Silver sulfide nanoparticles (Ag_2_S) act as a photosensitizer able to generate reactive oxygen species (ROS) after stimulation with a light source at a specific wavelength.

A target chemotherapy system was generated by combining the CD20 affinity of rituximab with the cytotoxicity of nab-paclitaxel (ABX). ABX is a form of paclitaxel, an antineoplastic agent, that has been chemically modified to bind albumin. This modification improves the drug's solubility and enhances its distribution to tumor tissues. The albumin scaffold was used to non-covalently conjugate ABX with rituximab (AR160), allowing both drugs to maintain their individual functions. AR160 showed improved anticancer efficacy in a Burkitts lymphoma xenotransplant model compared to the individual drugs. In vivo imaging analysis suggested that the enhanced efficacy was due to higher drug deposition in the tumor, mediated by the antibody interaction with the tumor-expressed ligand [55] (Figure 5, Table 2).

#### 4.2.2. NPs Targeting the BCL2 Pathway

BCL2 plays a critical role in the physio-pathological mechanisms of lymphomas. Targeting BCL2 is a therapeutic strategy used in treating lymphoma patients. An active targeting strategy for Bcl2 inhibition was developed using diatomite NPs carrying anti-BCL2 siRNA and engineered with the idiotype-specific peptide pA2036 that specifically recognized the hypervariable region of the surface immunoglobulin B-cell receptor (BCR) on A20 lymphoma cells. Idiotype determinants are unique to each clonal B cell population and distinguish tumors from healthy cells within the same individual. Diatomite NPs successfully released biologically active siRNA into the cytoplasm of lymphoma cells, leading to downregulation of BCL2 and subsequent cell death [58]. Liposomes were used to deliver a 24-base single-stranded phosphodiester DNA oligodeoxynucleotide (PNT100) designed to inhibit BCL-2 promoter activity. The effective decrease in BCL2 mRNA and protein expression associated with cell death was demonstrated in lymphoma cells upon NPs treatment [87]. The versatility and unique properties of NPs allow for functionalization of their structures to simultaneously bind multiple surface receptors overexpressed on cancer cells. NPs coated with the polysaccharide hyaluronic acid (HA) were conjugated with an anti-CD20 antibody to deliver siRNA targeting the BCL-2 oncogene. HA is a ligand of the CD44 receptor upregulated on lymphoma cells, which undergo internalization after ligand binding. This nanocomplex demonstrated anticancer activity in vivo, demonstrating improved NPs internalization into cancer cells and effectively downregulating BCL2 in lymphoma cells due to the prolonged circulation time of siRNA. The dual ligand-receptor interactions (CD20 and CD44) enhanced tumor targeting and improved siRNA internalization through simultaneous endocytosis of both receptors [88] (Figure 5, Table 2).

#### 4.2.3. NPs and Peptide-Based Therapy

Lipid nanostructures have been effectively employed as vehicles for delivering peptide-based therapeutics in lymphoma treatment. Peptide-based therapeutics utilize short chains of amino acids as active components to exert therapeutic effects through various mechanisms, including blocking protein-protein interactions, disrupting cellular signaling pathways, or modulating immune responses. Therapeutic peptides (Tat-POSH), capable of disrupting protein–protein interactions and inducing cell death in hematological cancers, were assembled into micelles together with a lymphoma-specific DNA aptamer used as a homing device. These micelles were stable in conditions that mimicked serum, and aptamer-specific cell interactions occurred after ten minutes of incubation, leading to lymphoma cell death [89] (Figure 5, Table 2).

#### 4.2.4. NPs and Photodynamic Therapy (PDT)

NPs were tested as photosensitizing agents in the photodynamic therapy (PDT) of lymphoma. Photosensitizing agents undergo a photochemical reaction under light irradiation that generates reactive oxygen species (ROS), highly reactive molecules that can damage cancer cells, ultimately leading to cell death. Silver sulfide nanoparticles, Ag_2_S NPs, specifically induced the accumulation of intracellular ROS in lymphoma cells under irradiation and induced significant disruption of energy metabolism [90] (Figure 5, Table 2).

**Table 2:** Summary of selected studies using NPs for the treatment of lymphoid cancers.

Type of NPs	Nanomaterial	Functionalization	Tumor	Action Mechanisms	Advantages
Carbon	Diatomite	pA2036 peptide; siBCL2	Murine B cell lymphoma	Ig-BCR	Specific internalization of NPs and improved stability of nucleic acids [58]
Metal	Gold	sgc8c aptamer; daunorubicin	T-ALL	PTK7 binding and daunorubicin-mediated inhibition of DNA biosynthesis	Improved efficacy with lesser amount of drug, reduced side-effects, less frequent administration, increased patient convenience and compliance [64]
	Silver	rituximab	CLL	ROS overproduction, mitochondrial apoptosis	Improved antileukemic effects as compared with free rituximab [74]
Polymeric	1,4-butanediol diacrylate, 4-amino-1-butanol	leukemia-targeting CAR genes	B-ALL	Delivery of CD19 CAR genes into circulating T cell nuclei	Improved CAR-T cells manufacturing [66]
	Chitosan	Zinc	T-ALL	Fas receptor activation	Easy manipulation and cost-effective advantages [68]
		Rituximab; siNRF2; cyclophosphamide	CLL	CD20 targeting and induction of ADCC, CDC and CDCC (rituximab); inhibition of DNA duplication (cyclophosphamide); ROS accumulation (siNRF2)	Enhanced efficacy as compared with free cyclophosphamide reached through combined antileukemic mechanisms [76]
Lipid	Dipalmitoyl phosphatidylcholine, cholesterol and PEG	anti-CD19 Ab; imatinib	B-ALL	Internalization of CD19 after antibody binding and imatinib-mediated BCR-ABL inhibition	Reduce off-target and off-tumor side effects of free imatinib. Effective in imatinib resistant cells [62]
	MC3, cholesterol, DSPC, and PEG-DMG	siRNA	CLL	Gene silencing	Improved RNA stability and cellular uptake [80]
	DOPC, 1,2-dimyristoyl-sn-glycero-3-phospho-l-serine, and 1′-rac-glycerol	BAT1; doxorubicin	CLL	CXCR-4/CXCL12 axis inhibition (BAT1), DNA interfering (doxorubicin)	Increased local doxorubicin release, inhibition of microenvironmental stimuli [81]
	PEG2000-SH	SN-38; T cells	Burkitt lymphoma	Infiltration of LNPs-decorated T cells into lymph nodal tumor niches and release of SN-38	Delivery of drugs within hard-to-reach area exploiting T cell homing capability [82]

## 5. Future Perspectives

Nanotechnology offers a promising opportunity to develop nanoagents that could improve the diagnosis and treatment of diseases in the future. Nanomaterials hold potential benefits for enhancing the detection and treatment of cancer cells, demonstrating essential requirements for in vivo applications. Nanoformulations of highly sensitive imaging contrast agents have been shown to improve magnetic resonance imaging (MRI) for monitoring and visualizing the biodistribution of cancer cells [91]. The optical and electrical properties of nanomaterials, such as metallic-based NPs, could enable the ultra-sensitive detection of specific proteins or DNA mutations associated with leukemia or lymphoma with high sensitivity, even at very low concentrations. Several nanomaterials, including metal-, polymer-, and carbon-based NPs, showed greater brightness and photostability compared to traditional fluorescent dyes and can be used for the multiplexed detection of multiple biomarkers simultaneously due to their narrow emission spectra. This could be particularly useful in diagnosing complex cancers, where multiple biomarkers need to be assessed to determine the disease stage and prognosis. Additionally, these nanomaterials can interact safely with biological systems without causing toxicity or immune reactions and can be easily cleared from the body, which are essential features for in vivo applications.

Although nanomaterials may have several benefits that can enhance diagnostics, they have not yet been widely employed in actual cancer detection for hematological lymphoid malignancies. Several barriers or issues associated with NPs need to be addressed in terms of delivery efficiency, circulating time, and toxicity [92]. To ensure the safe and sustainable use of nanomaterials in the medical field, several measures can be implemented, such as rigorous and comprehensive risk assessments to evaluate the potential hazards and risks associated with specific nanomaterials before their deployment in medical applications.

There are numerous clinical and preclinical studies demonstrating the benefits of nanotechnology in cancer treatment, but it is critical that these advances are clinically translatable. Nanomaterials offer the possibility to improve the solubility and stability of both hydrophobic and hydrophilic anticancer molecules, thus enhancing bioavailability, drug circulation time, drug payload, and controlled drug release in response to specific stimuli (pH or temperature changes), allowing for a sustained therapeutic effect. Additionally, nanomaterials have shown promise in overcoming drug resistance by enabling the encapsulation of multiple drugs for combination therapies and enhancing drug delivery. Despite the fact that NPs offer many benefits in the treatment of lymphoid disease, some challenges remain to be addressed. Further research is needed to optimize their long-term safety, potential toxicity, optimal dose range, frequency, and duration of NPs to achieve their therapeutic goals while minimizing adverse effects [93].

Furthermore, the complexity of nanomaterials poses challenges for regulatory approval. Variability in nanomaterial synthesis and functionalization can lead to inconsistencies in diagnostic and therapeutic results. Standardizing the production and functionalization processes is crucial for ensuring reproducibility and reliability across different laboratories and clinical settings. Ensuring consistent manufacturing processes and demonstrating safety across diverse patient populations are key hurdles that need to be addressed through large-scale clinical trials. Additionally, the production and scaling of nanomaterials can be expensive, potentially limiting their accessibility in low-resource settings. Finding cost-effective methods for large-scale production is essential for their widespread adoption [94].

Nanotechnology for the diagnostics and therapies of lymphoid tumors stands to gain huge ground in the near future, creating a highly manageable cancer landscape for patients and hematologists.

## 6. Conclusions

In the last few years, nanotechnologies have been providing new concepts and approaches with potential benefits in the diagnosis and therapy of lymphoproliferative disorders. Still, we are in the emerging era of nanotechnology, and there is limited evidence for the use of nanotools in the precision medicine of lymphoid malignancies. Despite this, in this review, we have highlighted the main applications of nanotechnologies in managing lymphoid tumors.

NPs have shown the potential to improve the detection limits of conventional assays used in the diagnosis, prognosis, and monitoring of lymphoid disorders. The enhanced specificity and sensitivity achieved with the use of NPs as nanosensors could be useful for the evaluation of MRD after treatments, thereby minimizing the risks of refractory disease and disease relapse.

The great versatility of NPs could potentiate the therapeutic approaches currently used in treating lymphoid disorders. Nanomedicine has demonstrated the ability to improve the cytotoxic features of several drug regimens, including chemotherapy, immunotherapy, and targeted therapies. This improvement is due to the unique properties of nanomaterials, which can enhance drug stability, targeting, and release into cancer cells, thereby minimizing off-target and side effects.

NPs-based approaches represent a paradigm shift in the diagnosis and treatment of lymphoid malignancies, offering revolutionary solutions that hold the promise of improving patient outcomes and advancing the field of oncology towards precision medicine. However, challenges such as biocompatibility, safety, pharmacokinetics, and biodistribution remain to be addressed for the widespread clinical translation of NPs-based diagnostic and therapeutic strategies. Continued interdisciplinary collaboration among researchers, clinicians, and biomedical industries is crucial for advancing the field and realizing the full potential of NPs against lymphoid malignancies.
